# Supplementary figures and images for: Identification of Regulatory Elements in Primary Sensory Neurons Involved in Trauma-Induced Neuropathic Pain
Source: Mol Neurobiol. 2023 Oct 4;61(3):1845–59. doi: 10.1007/s12035-023-03673-5 (PMC10896855; doi:10.1007/s12035-023-03673-5)

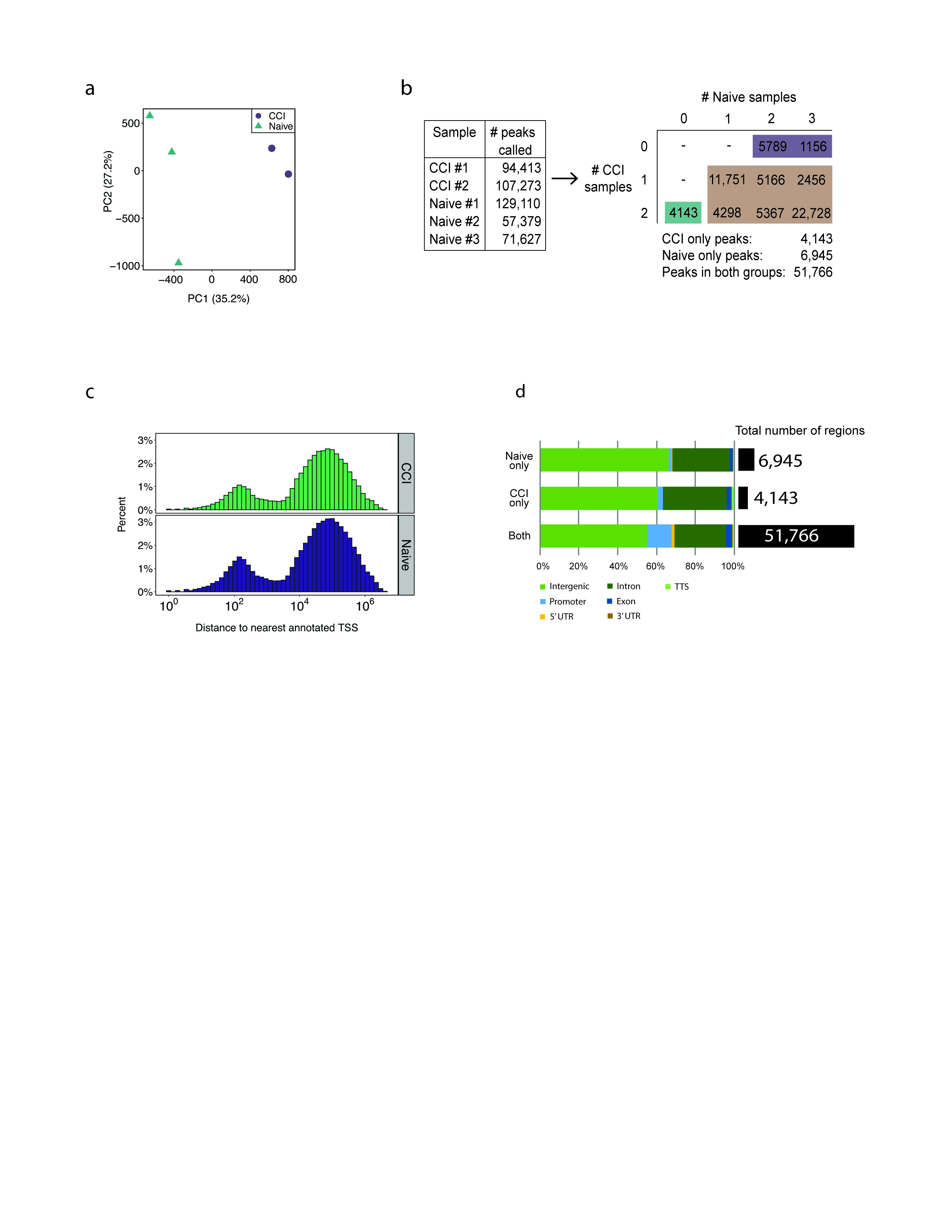

Supplement: Supplementary file 1 — ESM 1 [file 12035_2023_3673_MOESM1_ESM.tif]

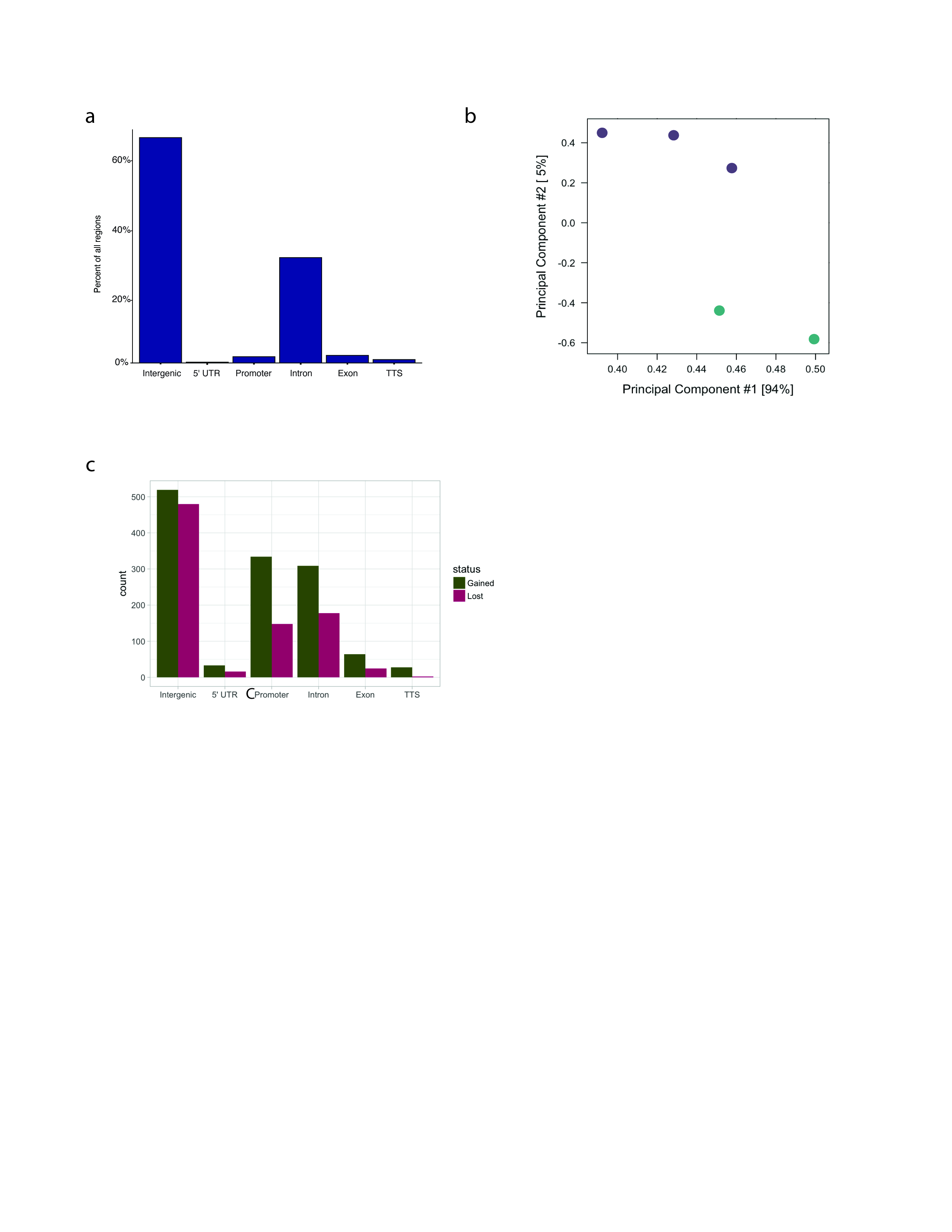

Supplement: Supplementary file 2 — ESM 2 [file 12035_2023_3673_MOESM2_ESM.tif]
